# The Buccal Fat Pad: A Unique Human Anatomical Structure and Rich and Easily Accessible Source of Mesenchymal Stem Cells for Tissue Repair

**DOI:** 10.3390/bioengineering11100968

**Published:** 2024-09-27

**Authors:** Gaia Favero, Cornelis J. F. van Noorden, Rita Rezzani

**Affiliations:** 1 Anatomy and Physiopathology Division, Department of Clinical and Experimental Sciences, University of Brescia, 25123 Brescia, Italy; rita.rezzani@unibs.it; 2 Interdipartimental University Center of Research “Adaption and Regeneration of Tissues and Organs (ARTO)”, University of Brescia, 25123 Brescia, Italy; 3 Department of Genetic Toxicology and Cancer Biology, National Institute of Biology, 1000 Ljubljana, Slovenia; c.j.vannoorden@nib.si; 4 Italian Society for the Study of Orofacial Pain (Società Italiana Studio Dolore Orofacciale—SISDO), 25123 Brescia, Italy

**Keywords:** buccal fat pad, human anatomy, mesenchymal stem cells source, oral surgery

## Abstract

Buccal fat pads are biconvex adipose tissue bags that are uniquely found on both sides of the human face along the anterior border of the masseter muscles. Buccal fat pads are important determinants of facial appearance, facilitating gliding movements of facial masticatory and mimetic muscles. Buccal fad pad flaps are used for the repair of oral defects and as a rich and easily accessible source of mesenchymal stem cells. Here, we introduce the buccal fat pad anatomy and morphology and report its functions and applications for oral reconstructive surgery and for harvesting mesenchymal stem cells for clinical use. Future frontiers of buccal fat pad research are discussed. It is concluded that many biological and molecular aspects still need to be elucidated for the optimal application of buccal fat pad tissue in regenerative medicine.

## 1. Introduction

Lorenz Heister, a German anatomist and surgeon, described the buccal fat pad for the first time in 1727 as a molar gland without specific physiological functions [1,2]. In 1801, the French anatomist Xavier Bichat defined this anatomical structure as an adipose tissue ball localized between the buccinator muscle, masseter muscle, and skin [3,4]. In 1977, Peter Egyedi reported, for the first time, possible surgical applications of the buccal fat pad as a pedicled flap to close oroantral and oronasal fistulae [5]. Finally, Tideman et al. [6] published a detailed anatomical description of the buccal fat pad in 1986. A series of patients were described who were surgically treated with uncovered buccal fat pad grafts underlining its clinical potential.

In the present review manuscript, the anatomy and morphology of buccal fat pads are briefly introduced to assist clinicians in the development of novel applications in reconstruction surgery as well as in regenerative medicine. Next, an overview of applications of buccal fat pad tissue in oral reconstructive medicine is provided, followed by optimization of buccal fat pad graft preparation procedures. 

The buccal fat pad is an anatomical structure with distinctive features that are different from features found in other fat compartments in face or body. Moreover, the buccal fat pad is an anatomically complex structure that has a huge impact on facial contour and appearance.

The anatomy of the buccal fat pad has been reported in various studies, but anatomical and lifetime-related variations and its boundaries have received less attention. In this introductory section, the buccal fat pad anatomy, including its boundaries, vascularization, and morphology, is discussed to underline the correlation with its increasing clinical significance. Furthermore, the intraoral approach to buccal fat harvesting is based on its anatomical feature, as is described in the following section. 

**Figure 1:**
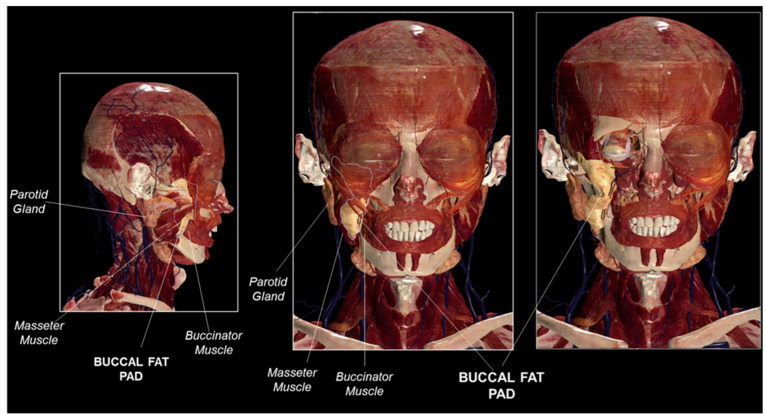
Buccal fat pad gross anatomy. Buccal fat pad anatomical localization and its relationship with landmarks in the face (such as parotid gland, buccinator muscle, and masseter muscle). Anatomage Inc. (Santa Clara, CA, USA)—Anatomage Table EDU. The 3D rendering of the body donated to scientific data is from Anatomage Table (the Head and Neck dataset).

The buccal fat pad is anatomically located along the anterior border of the masseter muscle, between the buccinator muscle at its medial side and the mandible at its lateral side, descending towards the retromolar region (Figure 1).

The buccal fat pad is covered by a thin connective tissue layer and is anatomically characterized by a main central body with four extensions, the buccal, pterygoid, pterygopalatine, and temporal extension [2,4,7,8,9,10,11]. The buccal extension is the major structure that determines shape, fullness and contour of the cheeks. 

The thick collagen-rich septum that originates from the external capsule divides the buccal fat pad into three lobes, which are closely related to nasolabial folds, anterior, intermediate, and posterior lobe [4,12,13,14,15,16], each with its own vascular supply and capsule [4,17,18]. 

**Figure 2:**
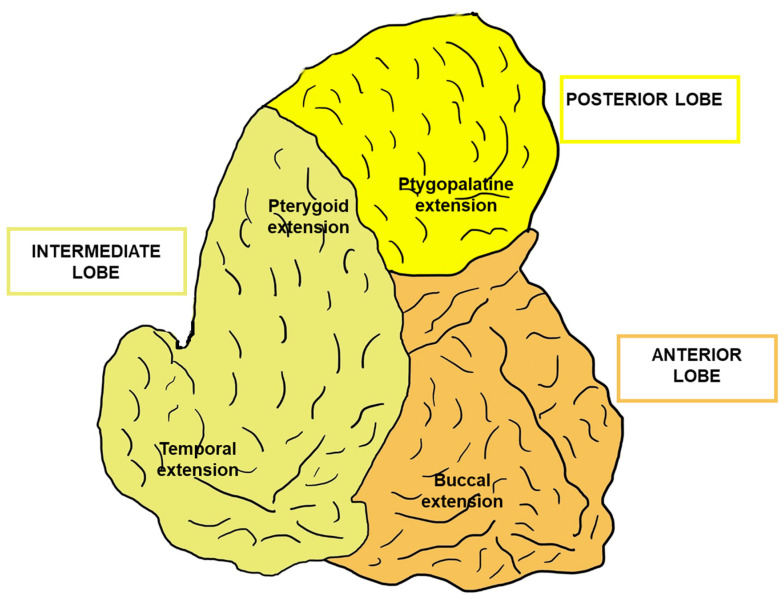
Buccal fat pad lobes and extensions. Schematic representation of the three buccal fat pad lobes (anterior, intermediate, and posterior lobes) and of the four buccal fat pad extensions (buccal, pterygoid, pterygopalatine, and temporal extensions). Illustration inspired by Loukas et al. [19].

Figure 2 shows the three lobes of the buccal fat pad and the four buccal fat pad extensions.

Buccal fat pads in men and women are similar. The mean volume of the buccal fat pad is 10.2 mL (range 7.8–11.2 mL) in males and 8.9 mL (range 7.2–10.8 mL) in females. The average thickness is 6 mm and the average weight is 9.7 g both in men and women [19]. The right and left buccal fat pad in both men and women are not necessarily symmetric, but have similar volumes [4,7,11,19,20].

The buccal fat pad has an abundant blood supply. The deep temporal artery, the buccal artery and the superior posterior alveolar artery are the three maxillary artery branches that sprout into a dense capillary network in the parenchyma of the buccal fat pad. Supplemental blood supply is provided by branches of the facial artery [7,13,21,22,23]. This abundant blood supply is tightly correlated with the high reparative potential of the buccal fat pad. Anatomical understanding of buccal fat pad vascularization is necessary to prevent undesirable events in the case of injuries during surgical approaches. Furthermore, the buccal fat pad flap is applied to reconstructed maxillary and oral defects because of its abundant blood supply [24].

The buccal fat pad venous drainage is provided by the facial vein [4,7]. 

**Figure 3:**
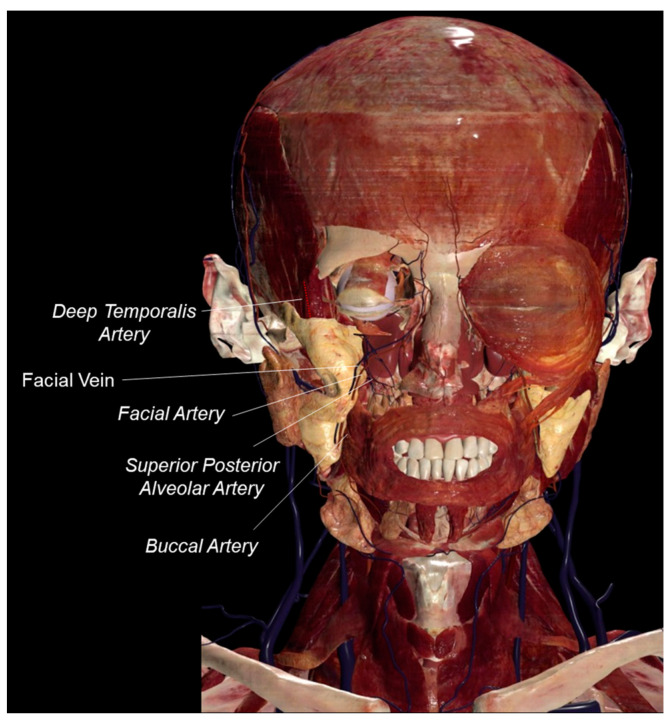
Buccal fat pad blood supply. The buccal fat pad blood supply is shown on the right side of the face after the removal of the masseter muscle, zygomatic major muscle, zygomatic minor muscle, orbicularis oculis, maxillary bone and jaw. The deep temporalis artery is indicated by a red dotted line to demonstrate that it runs underneath the temporalis muscle. Anatomage Inc. (Santa Clara, CA, USA)—Anatomage Table EDU. The 3D rendering of the body donated to scientific data is from Anatomage Table (the Head and Neck dataset).

Figure 3 shows the arterial branches that provide the buccal fat pad arteries’ blood supply and the buccal fat pad venous drainage.

A number of lifetime-related buccal fat pad anatomical changes have been described. The buccal fat pad is prominent in newborns and infants, and it is one of the earliest sites of well-developed fetal adipose deposition [2,15,25]. During a lifetime, the structure of the buccal fat pad changes, and its prominence relative to surrounding structures becomes smaller over time [15]. In particular, the buccal extension of the buccal fat pad reduces in size with age, thus decreasing support for the medial and middle cheek “components” [26,27]. Gierloff et al. [26] demonstrated in a post-mortem study that during aging, the downward migration of the facial fat compartments occurs, leading to a crescent-shaped cavity below the lower edge of the orbicularis oculi muscle and to the deepening of the nasojugal fold. These observations indicate that midface appearance during aging is not only related to gravity forces, but also to the buccal fat pad aging-related altered volume, with an important impact on facial aesthetics. However, it is essential to realize that, in contrast to other fat compartments, there is no correlation between the volume of the buccal fat pad and overall individual body weight or fat distribution [2,28,29].

The buccal fat pad functions as a gliding pad among masticatory and mimetic muscles, which facilitate the movement of one muscle relative to the other [2,4,11,25,30]. During chewing and speaking, the buccal fat pad preserves adjacent anatomical structures from the extrusion that may occur either due to muscle contraction or as a consequence of outer forces [2]. The buccal fat pad functions as a shock absorber during masticatory activity and protects facial neurovascular bundles that are present in the masticatory space [4,8,22,30,31]. Furthermore, the buccal fat pad has a fundamental function in infants as well during breast feeding. For this reason, the buccal fat pad is also named the “suction pad”. The buccal fat pad outbalances negative pressure on the cheek during suckling and increments the buccinator muscle function to prevent its collapse during breast feeding. Difficulties in suckling during the breast feeding of premature and ill-developed infants may be, at least in part, related to the improper development of the buccal fat pad [4,7]. 

## 2. The Buccal Fat Pad as a Stem Cell Source

Morphological evaluation of buccal fat pads has revealed the presence of a large amount of mesenchymal stem cells, especially in its most vascularized part, indicating the high reparative potential of this fat pad, which explains why it is a preferential choice option as an autogenous donor “material” in regenerative surgical interventions.

Mesenchymal stem cells from different sources show differences in marker expression; however, mesenchymal stem cells from the oral cavity show similar therapeutic potential in bone regeneration [31,32,33,34]. Notably, the buccal fat pad is not to be considered a mere anatomical structure, it may also be considered as one of the most suitable intra-oral sources of stem cells for use in tissue engineering; additionally, because this stem cell source is readily available, there are also important molecular developments in clinical applications [35,36,37].

**Figure 4:**
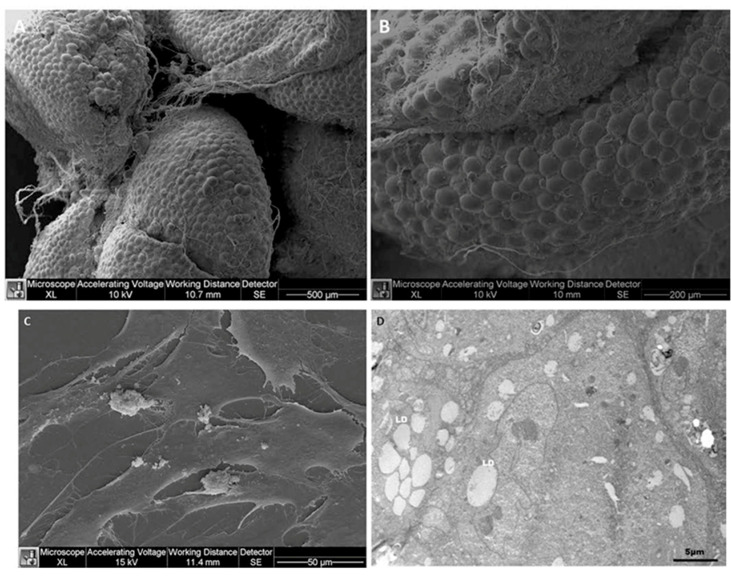
Scanning electron microscopical (SEM) images (**A**–**C**) and transmission electron microscopical (TEM) image (**D**) of adipocytes of a human buccal fat pad. In photomicrographs (**A**) and (**B**), mature adipocytes organized in lobules in the buccal fat pad are shown in low-magnification SEM images, and in photomicrograph (**C**), cultured mesenchymal stem cells harvested from the buccal fat pad are shown at higher magnification. The TNT-like long and thin cytoplasmic protrusions are visible both in situ (**A**,**B**) and in cultures of mesenchymal stem cells (**C**). The high-magnification TEM photomicrograph (**D**) of cultured mesenchymal stem cells shows intracellular small lipid droplets and an organelle-poor cytoplasm. (LD): lipid droplets. The photomicrographs are from Conti et al. [22].

Conti et al. [22] described the presence of mesenchymal stem cells with features typical of non-differentiated cells in the buccal fat pad. In fact, buccal fat pad-derived mesenchymal stem cells present ample cytoplasm, long protrusions, numerous lipid droplets, and relatively low amounts of organelles. The long and thin cytoplasmic protrusions may be tunneling nanotubes (TNTs) that function as communication pipelines between cells of the same type (for example pericytes) [38] but also between different types of cells (for example astrocytes and brain tumor cells) [39,40]. Both in the lobules of mature adipocytes in the buccal fat pad (Figure 4A,B) and in the cultured mesenchymal stem cells harvested from the buccal fat pad (Figure 4C,D), abundant amounts of long and thin cytoplasmic TNT-like protrusions are present. The function(s) of intracellular TNTs in the buccal fat pad is (are) obscure at present but may well be relevant for intracellular communication between adipocytes in general or in the buccal fat pad in particular. As far as we know, TNTs in fat compartments have not been studied yet. 

A subpopulation of mesenchymal stem cells was identified by the simultaneous expression of specific markers, SEEA3, and CD105, which classified this cellular subpopulation as mesenchymal stem cells defined as multilineage-differentiating stress-enduring cells that can differentiate into multilineage osteoblasts, chondroblasts, adipocytes, and other types of differentiated cells. These cells are defined as non-tumorigenic stress-tolerant and pluripotent cells with high regenerative potential and migration capacity into the damaged tissue [22,41,42,43]. Consequently, buccal fat pad mesenchymal stem cells are a favorable choice for regenerative medicine. Adipose-derived mesenchymal stem cells from buccal fat pads or from other parts of the body demonstrated similarities in cell yield, morphology, and multilineage differentiation. On the other hand, buccal fat pad-derived mesenchymal stem adipocytes have been reported to be slightly smaller, with a round shape as compared to subcutaneously harvested stem adipocytes [33]. However, unlike subcutaneous stem adipocytes, stem adipocytes derived from the buccal fat pad can form colonies in vitro, and the dedifferentiated fat cells are able to promote bone and periodontal tissue regeneration [29,43,44,45,46,47].

Buccal fat pad mesenchymal stem cells exhibit a larger clinical and bioengineering applicability due to the easy anatomical availability and accessibility of this fat pad. In line with these observations, Genova et al. [36] isolated dental pulp and the buccal fat pad of the same patients (12 healthy patients—7 males and 5 females, mean age 24 ± 3 years) and characterized the stem cells of both sources. These authors, for the first time, evaluated two stem cell populations derived from the same patients in a direct comparison to assess which was the most promising source of mesenchymal stem cells. It was observed that the buccal fat pad stem cells present a spindle-shaped morphology, as typically observed in mesenchymal stem cells, whereas dental pulp stem cells showed a polygonal shape, suggesting that cell morphology depends on the tissue source. Notably, the two cell populations were molecularly characterized by flow cytometry. Both buccal fat pad stem cells and dental pulp stem cells expressed markers of mesenchymal stem cells phenotypes, such as CD105, CD44, CD73, and CD90, whereas the expression of CD45, a marker of hematopoietic cells, according to the requirements proposed by the International Society for Cellular Therapy in defining mesenchymal stem cells, was lacking [41,48]. Specifically, approximately 80% of the buccal fat pad stem cells expressed the markers CD105, CD44, CD73 and CD90, while being negative for CD45, whereas only 18% of dental pulp stem cells expressed mesenchymal stem cell markers. These data confirm and outline the different mesenchymal stem cell yields from the different sources, which thus may influence stem cell retrieval and assist clinicians to develop novel applications in regenerative medicine. Furthermore, osteocalcin, the major bone non-collagenous protein synthesized by osteoblasts, odontoblasts and hypertrophic chondrocytes [49], was expressed a higher level in the buccal fat pad stem cells as compared to dental pulp stem cells [36]. To assess the early osteogenic differentiation of buccal fat pad stem cells and dental pulp stem cells, the transcription levels of the pro-osteoblastic genes collagen type I and runt-related transcription factor-2 (RUNX-2) [50] were evaluated. Both pro-osteoblastic genes were highly expressed in the osteodifferentiated cells obtained from both sources. Notably, buccal fat pad stem cells can grow and differentiate, and they outperform dental pulp stem cells. All these data are of fundamental importance when a choice is to be made about whether to employ extracted teeth or adipose tissue for bone regenerative protocols. Similar data were reported by Meshram et al. [51] in a clinical study in which the buccal fat pad-derived mesenchymal stem cells were characterized so confirming that buccal fat can be considered to be a valid tool for tissue engineering applications. However, the modulation of buccal fat pad stem cell differentiation into osteoblasts was difficult, and it is fundamental to remove the residual pluripotent stem cells with teratoma potential before clinical application [48,52]. Faster osseous regeneration and repair of bony defects through buccal fat pad stem cells as compared with normal bone healing procedures actually represent an important boost to patient’s overall health.

Furthermore, the buccal fat pad stem cells are applied for chondrogenic differentiation as well. Dehghani Nazhvan et al. [35] observed in an in vivo study that the preconditioned-hypoxic cultured buccal fat pad stem cells together with a bilayer chitosan scaffold can promote the regeneration of articular cartilage defects without using any chondrogenic growth factors. Interestingly, the authors reported that chondrogenic differentiation and osteochondral conjunction in articular cartilage defects via buccal fat pad stem cells-seeded bilayer scaffolds may be increased by hypoxic preconditioning. These observations may be related to both oxygen-sensitive transcription factors of hypoxia-inducible factor-1α and -2α (HIF-1α and HIF-2α), which both play critical roles in the chondrogenicity of stem cells. Apparently, this study provides new insights into the cartilage regeneration potency of preconditioned buccal fat pad stem cells.

Table 1 lists buccal fat pad mesenchymal cell studies published thus far and reports their major findings. These data open new horizons in the development of tissue engineering and its interdisciplinary interaction with various dental disciplines.

In summary, due to the buccal fat pad anatomo-morphological aspects, this fat pad is considered to be an important source of autologous stem/progenitor cells, which can be used for different clinical applications [32,60]. The buccal fat pad is a promising option among other autogenous donor “materials” in regenerative medicine and tissue engineering. This is relevant for maxillofacial and dental surgeons, as buccal fat pads are easily accessible. In addition, buccal fat pad mesenchymal stem cells can be converted into bone tissue, thus not only allowing for the repair of postoperative defects but also the restoration of a whole complex of lost tissues as well. However, it is important to underline that, despite the various potentials of buccal fat pad mesenchymal stem cells, the number and proliferative capacity of buccal fat stem cells are lower in old donors as compared to younger patients [32,51].

## 3. Relevance of the Buccal Fat Pad in Surgery

In recent years, clinical studies have been published that show that the buccal fat pad can be applied in oral reconstructive surgery as a versatile option. Further, buccal fat pad reduction or the bichectomy procedure (a surgical procedure that removes the buccal fat pad) can limit the appearance of a round face, can enhance zygomatic prominences, and can improve patients’ quality of life with respect to oral health and facial aesthetics satisfaction, thus promoting bichectomy as a “popular” technique in plastic surgery. To avoid the appearance of premature aging by plastic surgery, a detailed anatomical knowledge of the buccal fat pad, of its relationship with surrounding anatomical structures, and an exhaustive preoperative facial analysis are fundamental to adequately planning buccal fat pad excision [11,72]. Furthermore, bichectomy has a low morbidity, and complications are rarely reported [8,72,73,74,75,76]. 

More clinical studies are needed to optimize the surgical procedures of buccal fat pad reduction or removal and the clinical outcomes. Performing buccal fat pad reduction is a plastic surgery and aesthetic procedure, but it can cause various lesions in the noble anatomical structures located near the buccal fat pad. In order to prevent complications, adequate anatomical understanding is fundamental, as well as proper postoperative wound care and treatments. Furthermore, long-term follow-up studies are needed to evaluate the application of this surgical approach.

As described above, the buccal fat pad can be used for reconstructive surgery in various clinical situations to “treat” intraoral defects. In the last decade, various studies have evaluated the utility of the buccal fat pad in the areas of oral defect repair and trauma [75,76,77,78,79]. The buccal fat pad can be used as a pedicled flap or as a free graft. In fact, it is a thin and pliable flap that can provide a long and widely based pedicle. Buccal or retromandibular defects can be successfully reconstructed with the buccal fat flap positioned over the rich vascular bed of the muscles in the recipient area [21,80]. An advantage of this buccal fat pad application is that it may be mucosalized when used for oral cavity reconstruction [13,17,81,82,83]. It is important to realize that a fundamental factor for buccal fat pad graft success is the maintenance of the capsule and vascularity during the buccal pad fat mobilization [21]. However, a critical factor is the small size and volume of the buccal fat pad, which maximally provides 7 × 4 × 3 cm^3^ of tissue to be transferred and thus cannot be used for larger defects [15,84,85].

As previously reported, the buccal fat pad has many advantages over other types of flaps: anatomical location, rich blood supply, easy harvesting, and minimal dissection requirements [25,71]. To date, the surgical application of the buccal extension and body of the buccal fat pad are well documented; however, only few studies are available of the surgical use of other extensions of the buccal fat pad. Rathod et al. [86] proposed that in carefully selected cases, the temporal extension of the buccal fat pad may offer superior outcomes and should be considered as a viable option for the successful closure of large oro-antral communication defects (of up to 20 mm^2^ in size).

The buccal fat pad may also represent an important source of adipose tissue-derived stem cells to be utilized in maxillofacial or oral reconstructive surgery [4,87,88]. In fact, the buccal fat pad is an accessible source of stem cells that can be obtained easily via the oral cavity without injury to the external body surface. Furthermore, buccal fat pad-derived mesenchymal stem cells enable multilineage differentiation, proliferate quickly, and are more prone to producing colonies compared to adipose tissue-derived stem cells from other parts of the body [43]. Understanding of the molecular and regenerative potential of buccal fat pads may become a milestone for facial reconstructive and aesthetic surgery. In this context, the buccal fat pad represents an alternative source of adipose tissue, rich in pluripotent elements, which allow traumatic procedures of liposuction to be avoided [22].

Pilot clinical studies have applied the autologous buccal fat pad adipose tissue-derived stem cells to repair bone defects in the maxilla or mandible jaws [74] or for the augmentation of atrophic posterior mandibles in combination with the application of inorganic bovine bone mineral [65,69,75]. These clinical trials described successful procedures and new protocols for possible buccal fat pad applications. Specifically, Meshram et al. [51] conducted a pilot study on maxillofacial bone defects repaired by the application of buccal fat pad-derived mesenchymal stem cell grafts at the site of an enucleated jaw. The authors performed a single-stab incision on five patients (two males and three females; age range 18–55 years) in the depth of the vestibule opposite to the upper second molar and removed the buccal fat pad. Next, 5–10 mL of buccal fat pad was excised under aseptic conditions, and buccal fat pad cells were allowed to dedifferentiate in mesenchymal stem cell media and to transdifferentiate into osteoblast lineages and finally centrifuged to obtain a pellet. The pellet was then implanted into the patients. Despite the pilot character of the study, the small sample size, and the brief duration of the follow-up, the study confirmed that the buccal fat pad may be a useful “tool” for tissue engineering in oral and maxillofacial surgery. However, it must be realized that cultures of buccal fat pad-derived mesenchymal stem cells are rather expensive, an aspect that is considered to be a limitation.

After preliminary studies, Khojasteh et al. [65,67,69] evaluated in an exploratory clinical study the application of buccal fat pad-derived mesenchymal stem cells in the surgical repair of atrophic posterior mandibles. Fourteen patients (eight males and six females; age 41–65 years old) enrolled in this clinical study and underwent unilateral/bilateral horizontal/vertical augmentation procedures before implant insertion at the level of the posterior mandibular edentulous area. The authors compared the effects of buccal fat pad-derived mesenchymal stem cells associated with different graft material: anorganic bovine bone mineral or particulated autologous bone, considered, respectively, as “experimental” or “control”. For all the patients enrolled, the buccal fat pad was reached through a vestibular horizontal incision distal to the maxillary second molar. Then, 3–5 mL of buccal fat pad tissue was extricated in aseptic conditions prior to bone augmentation surgery. The buccal fat pad-derived mesenchymal stem cells were isolated from harvested buccal fat pads and combined with anorganic bovine bone mineral (at a 50/50 ratio) or with particulated autologous bone. It was found that bone regeneration occurred in both horizontal and vertical alveolar deficiencies in all patients and both graft materials associated with buccal fat pad-derived mesenchymal stem cells were efficient in mediating bone augmentation. However, limitations of this study were the low sample size, a follow-up of brief duration, and a lack of histological and histomorphometric analysis. Further studies to confirm these observations are needed; however, it is important to realize that the buccal fat pad is an interesting potential source of autologous stem cells and progenitor cells.

The advantages of oral reconstructive surgery applications of the buccal fat pad are (1) easy and convenient harvesting, (2) versatility, (3) few complications and (4) fast recovery. Moreover, during buccal fat pad collection, the occurrence of adjacent tissue traumatization is minimal. In fact, the procedure has a small donor-site morbidity, and the buccal fat pad can be applied in oral cavity reconstruction under local anesthesia [2,4,12,15,51,79]. The abundant blood supply facilitates a high take-up rate and quick epithelization of the buccal fat pad graft [89]. Moreover, the buccal fat pad is seldomly involved in cancer of the buccal mucosa, so this fat pad may represent a safe reconstructive technique in the early stages of cancer of buccal mucosa [21].

It is important to mention that the buccal fat pad has some limitations in reconstructive surgery, as previously reported. It can only cover small to medium-sized defects but not large defects. A major disadvantage of the use of the buccal fat pad as an autologous flap is that it can be used only once per patient [17,85,90]. Furthermore, the buccal fat pad flap application has to be avoided in patients who have been irradiated previously [15,21,30,80]. On the other hand, application of the buccal fat pad is a highly efficacious treatment option with satisfactory comfort and safety. It can be achieved relatively easily, and the buccal fat pad therefore has the potential to become one of the standard non-invasive choices in bioengineering and in reconstruction surgery.

## 4. Conclusions and Future Directions

Buccal fat pads have various clinical applications, and the present review discusses the surgical implications of the close association between buccal fat pads and surrounding anatomical structures. A thorough understanding of topographical anatomy and the assessment of potential surgical risks are essential for successful surgical interventions. Moreover, the buccal fat pad morphology is important for its application in regenerative medicine, and therefore, we provide an overview of applications of buccal fat pad tissue in oral reconstructive medicine, discuss the optimization of buccal fat pad graft preparation, and highlight the significance of the harvesting of mesenchymal stem cells from buccal fat pads for tissue engineering applications. It is concluded that a further understanding of buccal fat pad anatomy and morphology is needed to optimize its applications in regenerative medicine and in reconstruction surgery.

## Figures and Tables

**Table 1:** Buccal fat pad (BFP) mesenchymal stem cell (MSC) studies.

Reference	BFP MSC Key Findings
Arpornmaeklong et al. [53]	BFP MSCs promote bone regeneration and exhibited a higher proliferation rate than periodontal ligament stem cells
Etemadi et al. [54]	Osteogenic differentiation and expression of related genes are increased in BFP MSCs by the effects of a 635 nm diode laser without affecting cell proliferation
Homayouni et al. [55]	Osteogenic differentiation and expression of related genes are increased in BFP MSCs by the effects of 980 nm irradiation
Li et al. [56]	Optimized protocol of isolation of MSCs from BFP
Zhidkov et al. [57]	BFP MSCs are preferentially localized around capillaries and in brown fat tissue
Camacho-Alonso et al. [58]	BFP MSCs cultured on bioceramics improve bone regeneration in bone defects compared with bioceramics alone in healthy and osteoporotic rats
Gholami et al. [59]	Proliferation and osteogenic differentiation of BFP MSCs are increased by the effects of 940 nm irradiation
Dehghani Nazhvani et al. [35]	Hypoxia preconditioning of BFP MSCs in combination with a bilayer chitosan scaffold promote regeneration of articular cartilage defects in the absence of chondrogenic growth factors
Genova et al. [36]	BFP MSCs show a higher isolation rate than dental pulp MSCs and show higher expression of MSC markers and larger osteogenic differentiation capacity.
Khazaei e al. [60]	Large amounts of BFP MSCs are harvested and differentiate successfully into odontoblast-, osteoblast- and cementoblast-like cells.
D’Esposito et al. [61]	Hyperglycemia reduces BFP MSC growth and osteogenic differentiation potential, whereas platelet-rich plasma enhances their growth without impairing their osteogenic differentiation potential
Nokhbatolfoghahaei et al. [62]	BFP MSCs on a β-tricalcium phosphate scaffold highly express osteogenic markers
Akhlaghi et al. [63]	Human amniotic membranes loaded with BFP MSCs enhance bone regeneration and reduce bone resorption by creating a protective membrane
Hosseini et al. [64]	BFP MSCs are the best choice for bone tissue repair
Khojasteh et al. [65]	BFP MSCs in combination with anorganic bovine bone mineral ameliorate bone regeneration
Meshram et al. [51]	BFP MSCs express various adhesion molecules and are not cells of hematopoietic and angiogenic lineages. Maxillofacial bone defect repair by grafting BFP MSCs results in high bone density formation with enhanced bone trabecular formation, well-organized and well-vascularized lamellar bone with Haversian channels and osteocytes
Fang et al. [48]	BFP MSCs and periodontal ligament stem cells have a higher osteogenic potential than dental pulp stem cells
Ghaderi et al. [66]	BFP yields a greater proportion of MSCs than gingiva
Khojasteh et al. [67]	Phase I clinical trial shows that BFP MSCs and autogenous bone may enhance bone regeneration in alveolar cleft bone
Rezai Rad et al. [68]	BFP MSCs show higher proliferation rates and osteogenic capacities than adipose tissue- and bone marrow-derived stem cells. BFP MSCs express osteogenic and angiogenic markers
Khojasteh and Sadeghi [69]	BFP MSCs and autogenous bone increase osteogenic capacity and prevent graft resorption in patients
Tsurumachi et al. [70]	BFP MSCs show higher osteogenic differentiation capacity than BFP large dedifferentiated fat cells
Ardeshirylajimi et al. [71]	BFP MSCs show higher osteogenic differentiation capacity than adipose tissue MSCs and unrestricted somatic stem cells which is similar to that of bone marrow MSCs
Broccaioli et al. [47]	BFP MSCs are rather similar to subcutaneous stem cells in differentiation capacity, adherence behavior to biological and synthetic materials and osteogenic differentiation

## Data Availability

Not applicable.
